# Naturally Occurring Xanthones and Their Biological Implications

**DOI:** 10.3390/molecules29174241

**Published:** 2024-09-06

**Authors:** Ayodeji O. Oriola, Pallab Kar

**Affiliations:** 1 Department of Chemical and Physical Sciences, Faculty of Natural Sciences, Walter Sisulu University, Nelson Mandela Drive, Mthatha 5117, South Africa; aoriola@wsu.ac.za; 2 African Medicinal Flora and Fauna Research Niche, Walter Sisulu University, Nelson Mandela Drive, Mthatha 5117, South Africa

**Keywords:** xanthones, xanthone classes, biosynthesis, biological activities, structure–activity relationship

## Abstract

Xanthones are chemical substances in higher plants, marine organisms, and lower microorganisms. The most prevalent naturally occurring sources of xanthones are those belonging to the families Caryophyllaceae, Guttiferae, and Gentianaceae. Structurally, xanthones (9H xanthan-9-one) are heterocyclic compounds with oxygen and a γ-pyrone component. They are densely packed with a two-benzene ring structure. The carbons in xanthones are numbered from their nucleus and biosynthetic construct. They have mixed shikimate-acetate (higher plants) and acetate-malonate (lower organisms) biosynthetic origins, which influence their classification. Based on the level of oxidation of the C-ring, they are classified into monomers, dimers, and heterodimers. While based on the level of oxygenation or the type of ring residue, they can be categorized into mono-, di-, tri-, tetra-, penta- and hexa-oxygenated xanthones, bis-xanthones, prenylated and related xanthones, xanthonolignoids, and other miscellaneous xanthones. This structural diversity has made xanthones exhibit considerable biological properties as promising antioxidant, antifungal, antimicrobial, and anticancer agents. Structure-activity relationship studies suggest C-1, C-3, C-6, and C-8 as the key positions that influence the biological activity of xanthones. Furthermore, the presence of functional groups, such as prenyl, hydroxyl, glycosyl, furan, and pyran, at the key positions of xanthones, may contribute to their spectrum of biological activity. The unique chemical scaffolds of xanthones, their notable biological activities, and the structure–activity relationships of some lead molecules were discussed to identify lead molecules as possible drug candidates.

## 1. Introduction

**Figure 1:**
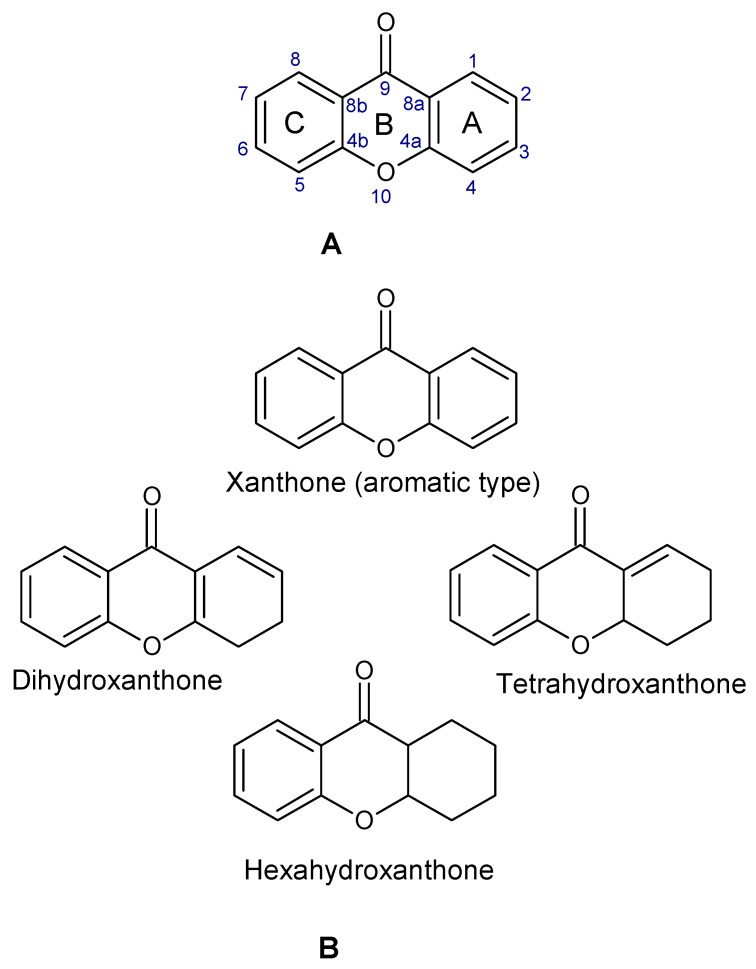
Chemical structure of xanthone, showing its (**A**) basic nucleus/tricyclic ring system and (**B**) the different oxidation states of the C-ring. The carbon numbers are indicated in blue color.

Xanthones are a heterocyclic class of secondary metabolites that are mostly found in lichen, fungi, and higher plant groups. They are formed from dibenzo-γ-pyrone, which is γ-pyrone condensed with two benzene rings (Figure 1A,B) [1,2]. The term “xanthone” was first used by J.C. Robert in 1961. Since these metabolites are typically formed as yellow solids, the word “xanthone” comes from the Greek word “xanthos”, which means yellow tint. The first documented xanthone derivative to be extracted from *Gentiana lutea* roots was gentian in 1821. The chemical formula of xanthone is C_13_H_8_O_2_, and its IUPAC designation is 9H-xanthen-9-one [3].

Lichens, fungi, plants (Polygalaceae, Moraceae, Gentianaceae, and Guttiferae families), and ferns all contain these tricyclic secondary chemicals. These metabolites are widely distributed in nature and because of their chemical makeup and position of the substituent groups on the aromatic ring, they have a variety of biological actions. Derivatives of xanthones come from two main sources: the marine environment (lower organisms) or naturally occurring sources of higher plants, which can be manufactured and extracted [4,5]. Researchers have been motivated to extract, separate, and purify these heterocyclic metabolites from their natural origins to prepare them as prospective candidates for drug development due to their unique structural architecture and known pharmacological effects. In the past twenty years, researchers have concentrated on understanding the structure–activity relationship of xanthones to use them effectively in medicine. Numerous xanthone derivatives, both natural and artificial, have been examined and found to offer major health benefits [6]. Xanthones and their derivatives can bind to several protein receptors involved in the etiology of diseases, making them have a wide range of biological activities, including antidiabetic, antioxidative, anti-inflammatory, anticancer, antibacterial, and antithrombotic effects [7,8,9,10,11,12].

There are six general categories into which xanthones can be categorized: prenylated xanthones, glycosylated xanthone, xanthonolignoids, oxygenated xanthone, xanthone dimers, and miscellaneous xanthones. Among them, oxygenated xanthones are divided into six subclasses based on the number of oxygen atoms they contain (mono-, di-, tri-, tetra-, penta-, and hexa-oxygenated xanthones). In addition, glycosylated xanthones are divided into two subclasses: C-glycosides (xanthones are associated with glycosyl moiety via carbon-carbon bond) and O-glycosides (glycosidic couplings among anomeric C-atom of sugar ring and O-atom of OH-group of xanthone structure) [13]. A diverse spectrum of biological actions is demonstrated by xanthone derivatives, both natural and synthetic. As secondary metabolites, xanthones are found naturally in lichens, fungi, various microorganisms, and higher plants [14]. Some of the plants, ferns, and fungus species that contain xanthones are *Artocarpus*, *Anthocleista*, *Allanblackia*, *Andrographis*, *Aspergillus*, *Bersama*, *Blackstonia*, *Calophyllum*, *Canscora*, *Centaurium*, *Chironia*, *Cratoxylum*, *Comastoma*, *Garcinia*, *Cudrania*, *Eustoma*, *Emericella*, *Frasera*, *Garcinia*, *Gentiana*, *Gentianella*, *Gentianopsis*, *Halenia*, *Hoppea*, *Hypericum*, *Ixanthus*, *Lomatogonium*, *Mesua*, *Morinda*, *Macrocarpaea*, *Mangrove fungi*, *Orphium*, *Peperomia*, *Pentadesma*, *Polygala*, *Penicillium*, *Phoma*, *Phomopsis*, *Rheedia*, *Rhus*, *Securidaca*, *Symphonia*, *Schultesia*, *Swertia*, *Tripterospermum*, *Vismia*, *Veratrilla*, and *Xylaria*. Currently undergoing a phase III clinical trial as an anticancer drug, 5,6-dimethylxanthone-4-acetic acid (DMXAA) is a noteworthy molecule with notable antibacterial and antitumor properties and provides a quick overview of its discovery [15]. The literature review unequivocally demonstrated that xanthones have a number of pharmacological activities, including anticholinesterase [16], α-glycosidase inhibitory activity [12], anticonvulsant [17], anthelminthic properties [18], anti-trypanosomiasis [19], anti-HIV [20], anti-hypertensive [21], anti-inflammatory [22], antimalarial [23], antibacterial [24], anti-enteroviral activity [25], antiprotozoal, antimicrobial and antioxidant activity [26], and antithrombotic activity [27]. Most notably, a number of xanthone derivatives have gained clinical appeal because of their molecular target locations on certain enzymes, including acetylcholinesterase, topoisomerase, *p*-glycoprotein, and α-glycosidase, as well as protein-protein interactions like p53-murine double minute 2 (MDM2) [28]. Though later withdrawn in 2014, the Food and Drug Administration (FDA, USA) approved amlexanox in 1996 for use as an anti-inflammatory, immunomodulatory (to treat stomach ulcers caused by aphthous ulcers), anti-allergic, and anticancer agent [29]. Thus, an attempt has been made to compile current and quantifiable data on a wide range of naturally occurring xanthone and derivatives and their biological implications in this review.

## 2. Methodology

The study involved an extensive literature search through various scientific databases (including Google Scholar, PubMedCentral, SciFinder, Scopus, and Web of Science) for information on naturally occurring xanthones. For the literature review, the following were the inclusive criteria: naturally occurring xanthones, history of xanthones, classes of xanthones, biosynthesis of xanthones, biological activities of xanthones (antifungal, antibacterial, anticancer, coagulant, antioxidant, anti-inflammatory, and anti-HIV/AIDS effects), and structure–activity relationship of xanthones. Exclusive criteria included a search for classes of compounds other than xanthones. All chemical structures were drawn using ACD/ChemSketch (Freeware) version 2021.1.1 (Advanced Chemistry Development, Inc., Toronto, ON, Canada).

## 3. History of Xanthones

De Koning and Giles discovered bikaverin, a wine-red pigment that was separated from many species of the fungi *Fusarium*, *Gibberella*, and *Mycogone*, in 1988 [30]. Bikaverin contains a quinone moiety, which may be responsible for its biological properties, such as antiprotozoal and antitumor activities [31,32]. The first report of natural xanthone, Gentisin (1,7-dihydroxy-3-methoxyxanthone), came from the higher plant *Gentiana lutea* in 1821, while the first prenylxanthone derivative, tajixanthone, was isolated from the fungus, *Aspergillus stellatus* in 1970 [33].

**Figure 2:**
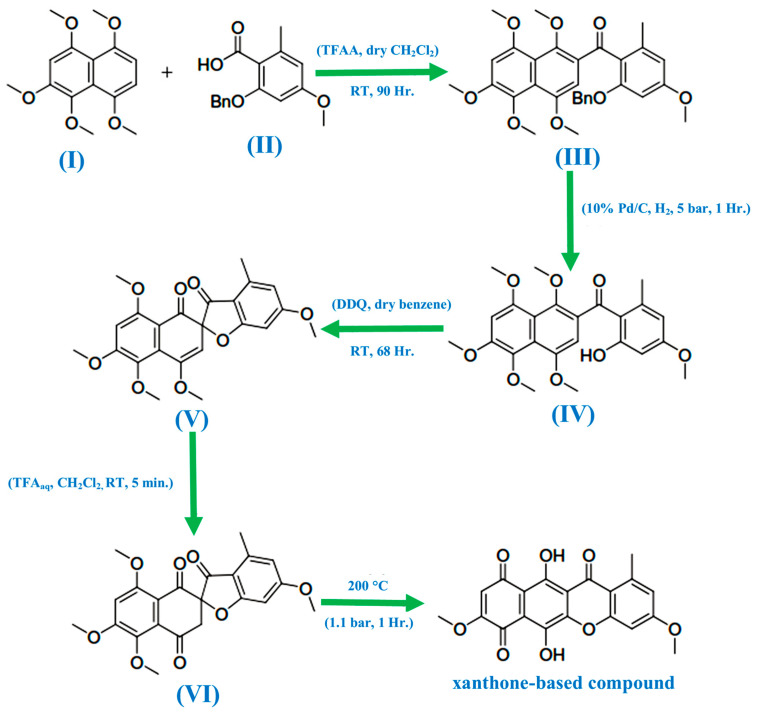
Reaction scheme to produce xanthone-based compounds [34].

In 1971, Bikaverin and norbikaverin were discovered by Kjaër and associates [34]. Using trifluoroacetic anhydride (TFAA) in combination with the synthesized naphthalene derivative (**I**) and the aryl acid (**II**), de Koning et al. [30] first introduced the carbonyl bridge to form the xanthone nucleus (Figure 2). This resulted in the intended product being produced as a single regioisomer (**III**) in a 51% yield. Palladium on carbon in the presence of hydrogen under pressure was then used to deprotect the compound, affording the phenol (**IV**) an 80% yield. Using 2,3-dichloro-5,6-dicyanobenzoquinone (DDQ) to oxidize the phenol (**IV**) resulted in the surprise production of the spiro compound (**V**) in a 61% yield. With the use of aqueous trifluoroacetic acid (TFA), the spiro compound was hydrolyzed to the required trione (**VI**) in a 94% yield. Pyrolytic isomerization of the trione (**VI**) resulted in a 93% yield of the xanthone-based chemical. The mechanism of the DDQ-facilitated reaction that results in the creation of the spiro compound has not been thoroughly explored since this synthesis. Investigating this innovative synthesis’s versatility was also necessary, especially regarding more electron-poor precursors. Owing to the wide diversity of biological activities exhibited by xanthones, it is critical to identify a flexible synthetic strategy that can support a variety of xanthone ring structures.

## 4. Natural Abundance, Classification, and Biosynthesis of Xanthones

From the natural source point of view, over 2000 xanthones have been reported from marine organisms, the lower and higher plants [34,35]. Higher plants are the most common sources of this unique group of compounds, comprising over 20 plant families, including Gentianiaceae, Guttiferae (Clusiaceae), Hypericaceae, Moraceae, and Polygalaceae, and over 120 species [14]. Among the common higher plants with xanthones are species of *Andrographis*, *Anemarrhena*, *Anthocleista*, *Calophyllum*, *Canscora*, *Caraipa*, *Garcinia*, *Gentiana*, *Hypericum*, *Kielmeyera*, *Mangifera*, *Mesua*, *Ochrocarpus*, *Peperomia*, *Polygala vulgaris*, *Rhus*, and *Swertia* [14].

Xanthone is structurally described as 9H-xanthen-9-one, a heterocyclic compound having a dibenzo-γ-pyrone moiety, with a basic molecular formula of C_13_H_8_O_2_ [3,35]. They are categorized based on their structural characteristics as xanthone monomers and xanthone dimers/heterodimers, and further into four subclasses based on the level of oxidation of the xanthone C-ring: fully aromatic-, dihydro-, tetrahydro-, and hexahydroxanthones (Table 1). The xanthone nucleus is numbered according to the mixed biosynthetic origins of the carbons in plants, which is in line with the IUPAC recommendations [36].

**Figure 3:**
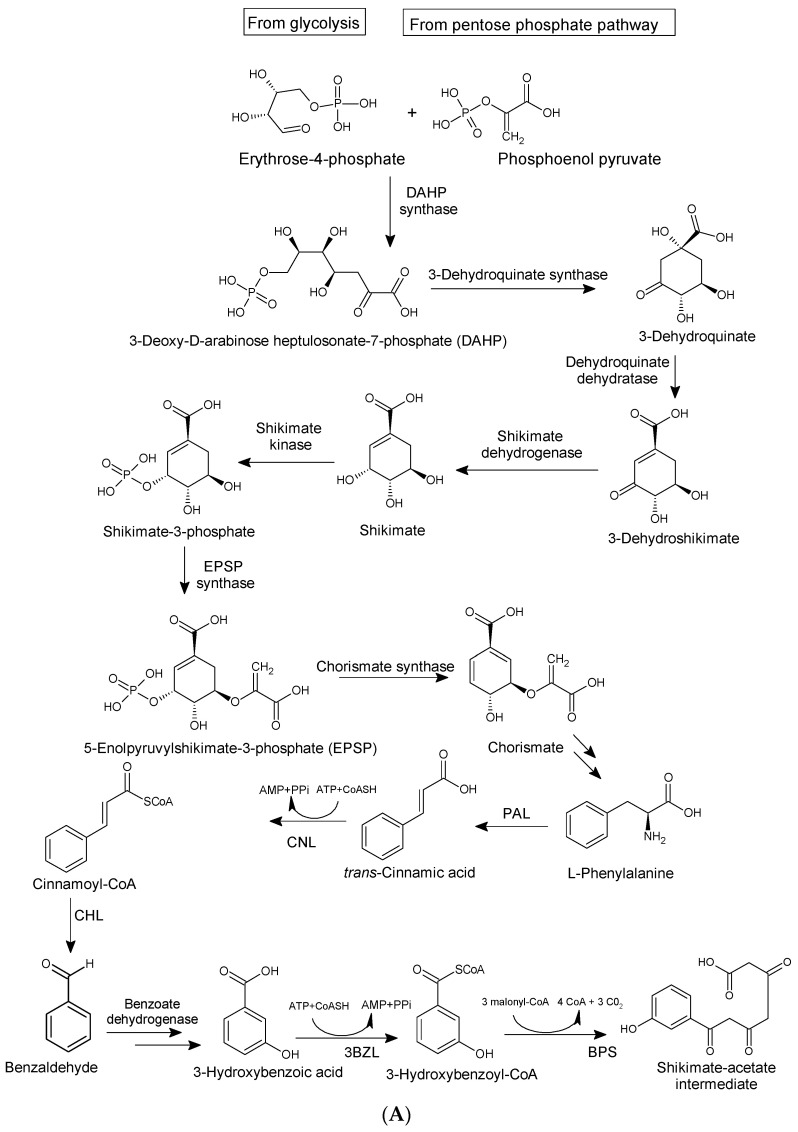

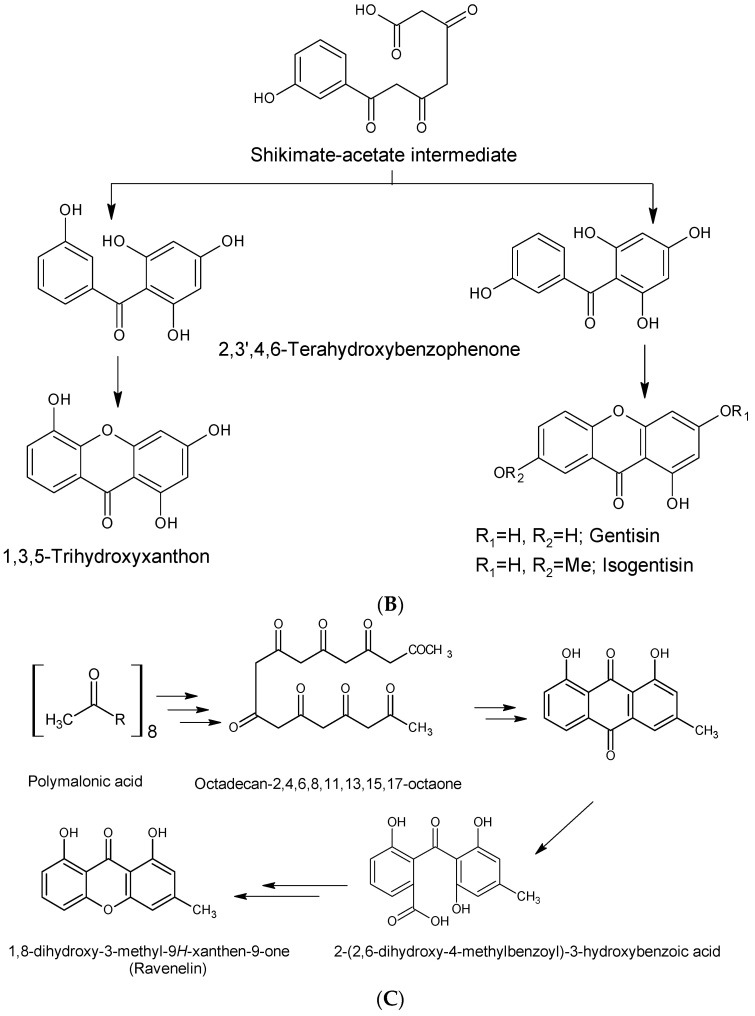
Biosynthetic pathways for xanthones via mixed shikimate-acetate origin (**A**,**B**) in higher plants [14] and acetate origin (**C**) in lower organisms [3].

Biosynthetically, xanthones from higher plants are derived from mixed shikimate-acetate origin [14], while those from fungi and other lower organisms are often acetate-derived [3,36], as presented in Figure 3A,B. In higher plants, the xanthone nucleus is of mixed biosynthetic origin, with the A- and C-rings giving rise to acetate and shikimic acid pathways, respectively (Figure 3A). The biosynthetic pathways for eliciting xanthones from *Gentiana lutea* are presented in Figure 3A,B. Here, 3-hydroxybenzoic acid derived from phenylalanine is coupled with three acetate units to form a polyketide. Aromatization of the side chain occurs, leading to a freely rotating benzophenone intermediate, which undergoes divergent oxidative phenolic coupling to give two different products, the 1,3,7-trihydroxyxanthone and the 1,3,5-trihydroxyxanthone mediated by xanthone synthase, a membrane-bound enzyme associated with cytochrome P450 [60]. In lower organisms, xanthones are biosynthesized from eight acyl groups, connected to each other to form 1,3,5,7,9,11,13,15-octaketonic intermediate. The octaketonic intermediate can cyclize to form benzoquinone and benzophenone intermediates. The benzophenone intermediate further transformed to produce ravenelin [3] (Figure 3C).

## 5. Biological Activities of Natural Xanthones with Structure–Activity Relationship (SAR) Insight

Xanthones have been linked to a number of biological characteristics, including antifungal, antibacterial, anticancer, coagulant, antioxidant, anti-inflammatory, anti-HIV/AIDS, and insecticidal effects.

### 5.1. Antifungal Activity

**Figure 4:**
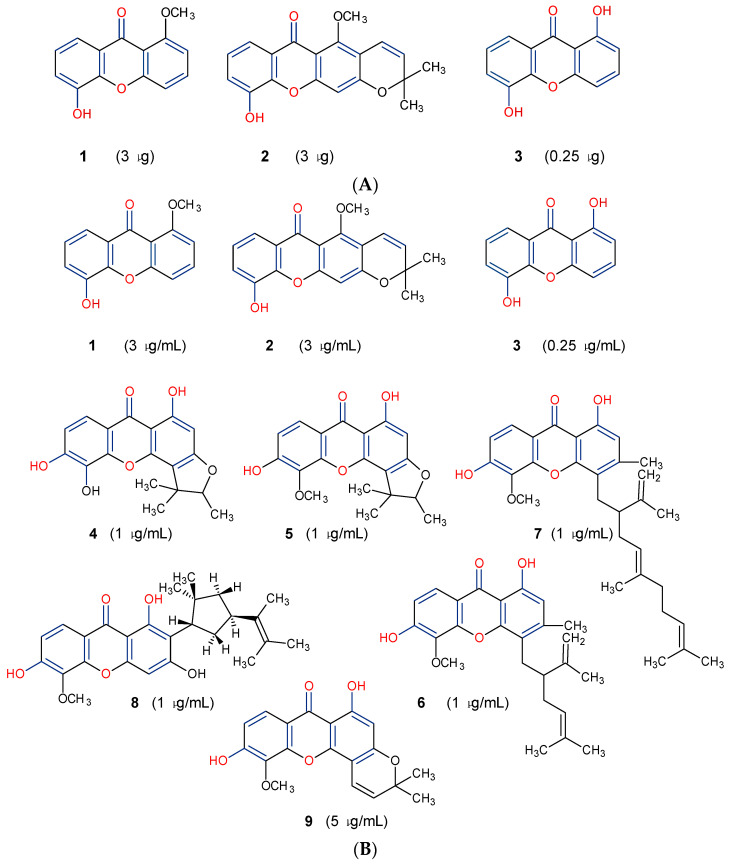
Antifungal activity of some naturally occurring xanthones showing the structure–activity relationships (colored moieties) against (**A**) *Cladosporium cucumerinum*; and (**B**) *Candida albicans* [61,62].

Three xanthones isolated from the dichloromethane extract of *Hypericum brusiliense* stems and roots, 5-hydroxy-1-methoxyxanthone (**1**), 6-deoxyjacareubin (**2**), and 1,5-dihydroxyxanthone (**3**), have been reported to demonstrate notable inhibition against the activity of the pathogenic fungus, *Cladosporium cucumerinum*. Based on the thin-layer chromatography (TLC)–bioautographic assay method, the minimum amounts of compounds **1**–**3** needed to inhibit the growth of the fungus were 3, 3, and 0.25 μg/mL, respectively, while propiconazole (standard drug) showed activity at 0.1 μg/mL [61]. Considering the structural configuration of these xanthones, it may not be far-fetched to attribute their considerable antifungal activities to the tricyclic xanthone nucleus and complete oxidation (aromatization) of the C-ring. Additionally, the presence of 1,5-dihydroxyl group in **3** might have contributed to its best activity, while an extra pyran ring seemed to have caused a notable reduction in the activity of **2** (Figure 4A). In another related study using the TLC bioautographic method, the antifungal activity of six xanthones, 2-deprenylreediaxanthone B (**4**), 5-O-methyl-2-deprenylrheediaxanthone B (**5**), calcinoxanthone D (**6**), roeperanone (**7**), 5-O-demethylpaxanthonin (**8**), and 5-O-methylisojacareubin (**9**), isolated from *H. roeperanum*, was evaluated against *Candida albicans* and *Cladosporium cucumerinum* [62]. The xanthones **4**–**8** showed selective inhibitory activity against *Candida albicans* at a minimum of 1 μg/mL, which was comparable to amphotericin B (standard drug = 1 μg/mL) but lower in activity than miconazole (standard drug = 0.001 μg/mL). However, the least active xanthone was **9**, requiring a minimum of 5 μg/mL for inhibition [62]. By structurally relating these xanthones to their antifungal activities, again, one could attribute the least potency of **8** to its extra pyran moiety (Figure 4B). On the other hand, the xanthone nucleus with a hydroxyl group at positions C-1 and C-6 might have contributed majorly to the similar antifungal activity displayed by **4**–**8**, while the presence of a furan ring attached to the C-ring of **4** and **5**, prenyl (lavandulyl) substituent at position C-4 of the xanthones **6** and **7**, as well as the cyclized terpene moiety attached to position C-2 of **8**, might not have contributed much to the antifungal activity of the respective xanthones (Figure 4B). It is worthy of mention that the presence of γ-pyrone and phenolic hydroxyl moieties as characterized by flavonoids and xanthones, enhances antimicrobial activity by increasing the hydrophilicity of the pathogen cell to cause leakage of ions out of the cytoplasm, leading to increased osmotic pressure in the cytoplasm, thereby resulting in cell lysis [63]. 

Future studies may include evaluating the potency of 1,5-dihydroxyxanthone (**3**) and that of xanthones **4**–**8** on clinical isolates of fungi, elucidating their molecular mechanisms of action, and obtaining more active analogs that can be optimized as antifungal drug candidates.

### 5.2. Antibacterial Activity

**Figure 5:**
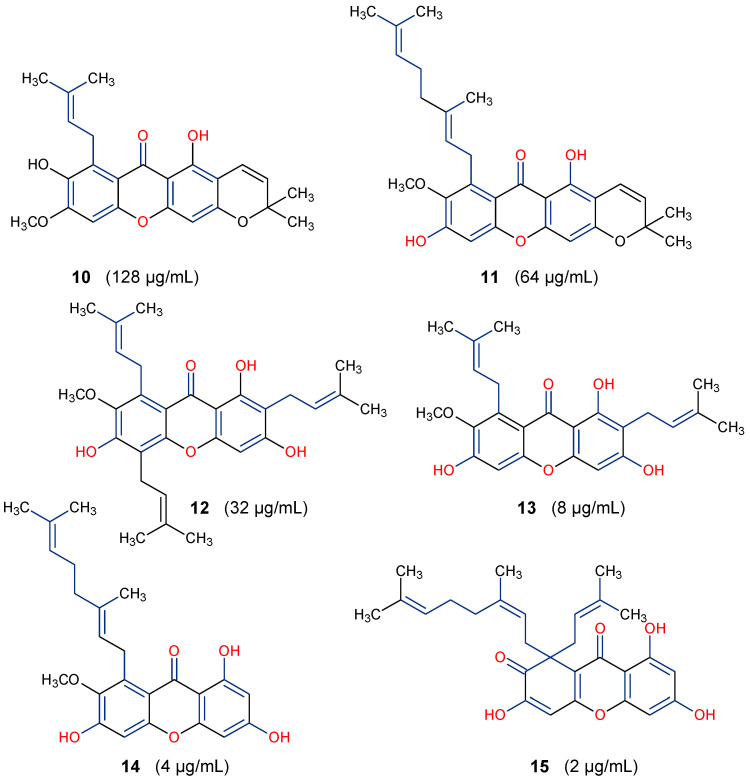
Antibacterial activity of some xanthones of *Garcinia cowa* against *Bacillus cereus* [64]. The bioactive moieties are illustrated in red and blue.

Some natural xanthones have been reported to show broad-spectrum antibacterial activities. For instance, the young fruits and flowers of *Garcinia cowa* afforded nineteen xanthones, with antibacterial activity exhibited against *Bacillus cereus*, *B. subtilis*, *Staphylococcus aureus*, *S. typhimurium*, *Escherichia coli* and *Pseudomonas aeruginosa* at a minimum inhibitory concentration (MIC) range of 2–128 µg/mL [64]. All 19 xanthones showed complete oxidation, that is, their C-ring was aromatized. However, based on the SAR study, six of the xanthones: garciniacowone E (**10**), fuscaxanthone A (**11**), *7-O*-methylgarcinone E (**12**), α-mangostin (**13**), rubraxanthone (**14**), and garcinianone B (**15**), showed varying level of activity, as presented in Figure 4. It was revealed that prenylation and oxygenation of the xanthone’s dibenzo-γ-pyrone core influence their antibacterial activity. There was an improvement in the activity of α-mangostin (**13**) against *B. subtilis* (MIC = 8 μg/mL) compared to **10** (MIC = 128 μg/mL). This could be attributed to the presence of prenyl substituent at positions C-2 and C-8 as well as the tri-oxygenation (trihydroxy substituent) at positions C-1, C-3 and C-6. Furthermore, the best activity (MIC = 2 μg/mL) displayed by garcinianone B (**15**) could be linked to the presence of a geranyl (C_10_) unit at position C-8 and the 1,3,6-trihydroxy substituent. Conversely, the presence of an extra pyran ring depleted the antibacterial activity, as revealed in garciniacowone E (**10**) with MIC of 128 μg/mL (Figure 5). Previous studies have shown that in addition to the ion-solubilizing properties of the phenolic groups, prenylation of xanthones and other flavonoid derivatives may help to alter the hydrophilicity–lipophilicity properties of the peptidoglycan lipid bilayers of the periplasmic membrane of the bacteria, making the membrane permeable for cell lysis [63,65]. In summary, the chemical diversity of xanthones provides an opportunity to elucidate their SARs, highlighting the importance of prenylation (geranyl) at position C-8 and hydroxylation at C-6 for optimal antibacterial activity. However, in vivo studies are required to determine further the safety and efficacy of lead xanthones such as rubraxanthone (**14**) and garcinianone B (**15**) for possible drug candidates against bacterial infections in the future.

### 5.3. Anticancer Activity

**Figure 6:**
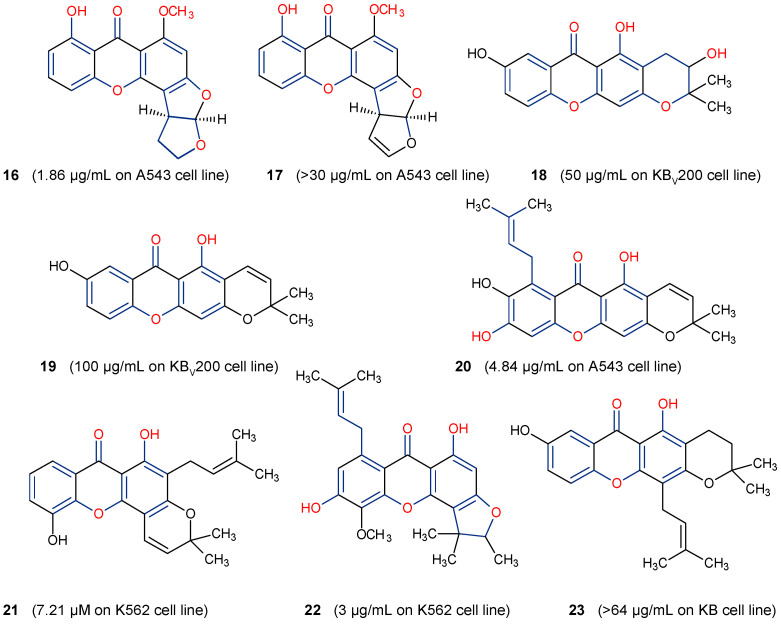
Some natural anticancer xanthones show the structure–activity relationships in red and blue.

Some natural xanthones have been reported to show selective cytotoxicity against human cancer cell lines. *Aspergillus versicolor* is the source of sterigmatocystin (**16**) and dihydrosterigmatocysin (**17**) [66]. These xanthones proved to be cytotoxic against HCT-15, SK-OV-3, A549, XF-498, and SK-MEL-2 cancer cell lines at 50% inhibitory concentration (IC_50_) range from 1.22 to above 30.00 µg/mL, with the former exhibiting more in vitro cytotoxicity than the latter. Additionally, **16** exhibited better cytotoxicity against Bel-7402 and NCI-H460 cancer cell lines than **17** [66]. The displayed anticancer activity of sterigmatocystin (**16**), for instance, an IC_50_ of 1.86 µg/mL against human lung cancer cell line A549, may partly be ascribed to its tetrahydrofuran substituent, unlike dihydrosterigmatocysin (**17**) with a dihydrofuran substituent (Figure 6). In another related study, the endophytic fungus ZSU-H16 isolated from the leaves of the mangrove tree *Avicennia marina* from the South China Sea coast afforded two xanthone derivatives, 3,5,8-trihydroxy-2,2-dimethyl-3,4,4-trihydro-2H,6H-pyrano[3,2-b]xanthen-6-one (**18**) and 5,8-dihydroxy-2,2-dimethyl-2H,6H-pyrano[3,2-b] xanthen-6-one (**19**) [67]. They both showed notable in vitro cytotoxicity against KB and KB_v_200 cancer cell lines. However, **18** exhibited better anticancer activity than **19**, with an IC_50_ value of 50 µg/mL against KB and KB_v_200 cancer cell lines [67]. These xanthones are related in structure, with both having an extra pyrano moiety. However, the higher activity of **18** may be due to the lower oxidation state of its pyrano moiety in addition to its more hydroxyl substituents (Figure 6).

A prenylated xanthone, 7-*O*-demethyl mangostanin (**20**) isolated from the pericarp of *G. mangostana* was also showcased in another study to exhibit broad-spectrum cytotoxicity against seven different cancer cell lines, U-87, SGC-7901, PC-3, H490, A549, CNE-1, and CNE-2 [68]. This xanthone demonstrated moderate to high apoptotic-dependent anticancer activity with IC_50_ values of 6.39, 8.09, 6.21, 7.84, 4.84, 3.35, and 4.01 µM, respectively. [68]. The study findings underscore the importance of prenylation of the xanthone core at position C-8 and oxygenation (hydroxyl substituent) at positions C-1, C-3, and C-6 to the biological activity of xanthones (Figure 5 and Figure 6). Two anticancer xanthones, Ananixanthone (**21**) and caloxanthone B (**22**) were isolated from the stem bark of *Calophyllum pressinervosum* [69]. Each xanthone exhibited considerable in vitro cytotoxicity, with IC_50_ values of 7.21 and 3.00 µM, respectively, against the K562 (leukemia) cell line. The better anticancer activity of **22** may be attributed to its prenylated substituent at position C-8, a furan ring attached to the C-ring, and hydroxyl group at positions C-1 and C-6. This is unlike in **21** where prenylation occurs at position C-2 with a pyran ring attached to the C-ring (Figure 5). A similar prenylated xanthone, dihydrobrasixanthone B (**23**) but with prenyl substitution at position C-4, was isolated from *Lisotrigona furva* [70]. Dihydrobrasixanthone B (**23**) displayed low in vitro anticancer activity with an IC_50_ above 64 µg/mL, which suggests that prenylation at position C-4 may have a diminishing effect on the anticancer activity of xanthones (Figure 6). These reports further underscore the significance of prenylation at position C-8 and furan ring attachment to the C-ring on the anticancer properties of xanthones.

**Figure 7:**
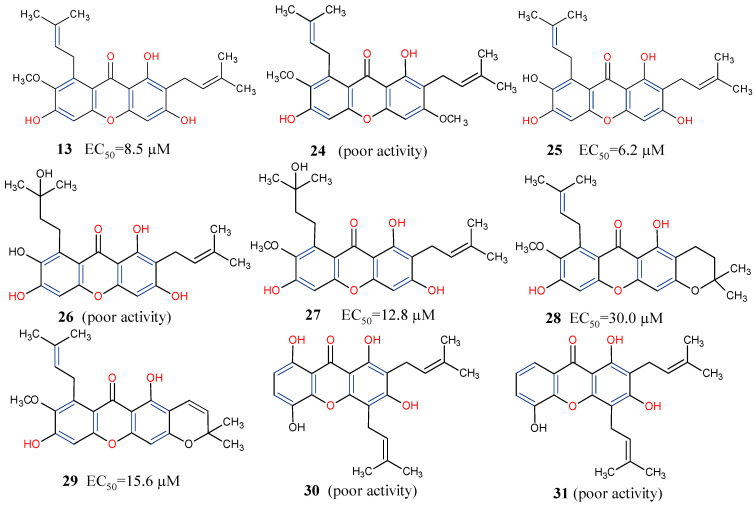
Some xanthones from *Garcinia mangostana* showing their structure–activity relationships based on CDK4 inhibition [77]. Functional groups in blue and red influence anticancer activity.

Lastly, about 70 different xanthones have been reported from the purple mangosteen, *Garcinia mangostana* [71,72,73,74]. Given the importance of xanthones among the heterocyclic compounds [75,76], and coupled with their potential as promising anticancer molecules, an activity-guided study of the plant led to the isolation of nine mangosteen xanthones, α-mangostin (**13**), β-mangostin (**24**), γ-mangostin (**25**), garcinone C (**26**), garcinone D (**27**), 9-hydroxycalabaxanthone (**28**), 3-isomangostin (**29**), gartanin (**30**), and 8-desoxygartanin (**31**). These compounds were further investigated through SAR for cyclin-dependent kinases (CDKs) inhibition [77]. It was revealed that prenylation at position C-4 rather than at C-8 (**30** and **31**) may not promote good CDK4 inhibitory activity (Figure 7). This, perhaps, might be due to the inability of the xanthones to bind to the ATP binding pocket of CDK4. Conversely, xanthones with an isoprenyl substituent at C-8 and hydroxyl group at positions C-1, C-3, and C-6 provided the most potent among the three xanthones, α-, β- and γ-mangostins (**13**, **24** and **25**). Moreover, when comparing γ-mangostin (**25**) to garcinone D (**27**), it is evident that when the isoprenyl moiety is hydroxylated, it can counter the negative influence of the methoxy group at position C-7 to restore the inhibition of CDK4 [77]. Further studies may include characterizing the key functional groups needed for specific pharmacological actions of γ-mangostin for lead optimization.

### 5.4. Antioxidant Activity

**Figure 8:**
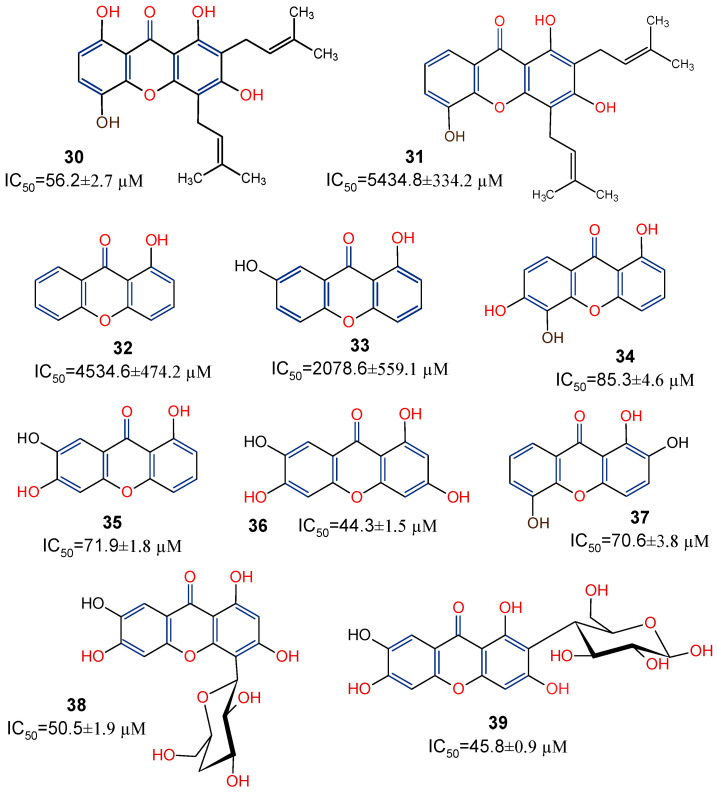
Xanthones from *Polygala japonica* with their in vitro ferric-reducing antioxidant power [78]. Functional groups indicated in blue and red are presented to show the structure–activity relationship.

Studies have shown the considerable antioxidative properties of natural xanthones. Ten xanthones: gartanin (**30**), 8-desoxygartanin (**31**), 2-hydroxyxanthone (**32**), 2,7-dihydroxyxanthone (**33**), 1,5,6-trihydroxyxanthone (**34**), 1,6,7-trihydroxyxanthone (**35**), norathyriol (**36**), 1,2,5-trihydroxyxanthone (**37**), isomangiferin (**38**), and mangiferin (**39**), were isolated from *Polygala japonica* and potentiated for antioxidant activity. Based on the ferric-reducing antioxidant power assay method, these compounds showed electron-transfer (ET) antioxidant potentials [78]. Further SAR analysis (Figure 8) suggests that the ET potential of the xanthones may reside within the xanthone’s dibenzo-γ-pyrone nucleus, with 1,3,6-trihydroxy substituent further enhancing the antioxidant activity, as shown in norathyriol (**36**). It was also revealed that, while prenylation at position C-2 may not play any major contributory role to the activity, a prenyl substituent at position C-4 may, on the other hand, deplete antioxidant activity, as shown in 8-desoxygartanin (**31**). The antioxidant capacity of isomangiferin (**38**) and mangiferin (**39**) provide more evidence to suggest that, rather than a prenyl substitution of the C-ring, the presence of sugar residue *(C*-glycosylation) may enhance the antioxidant capacity of xanthones [78].

### 5.5. Anti-Inflammatory Activity

**Figure 9:**
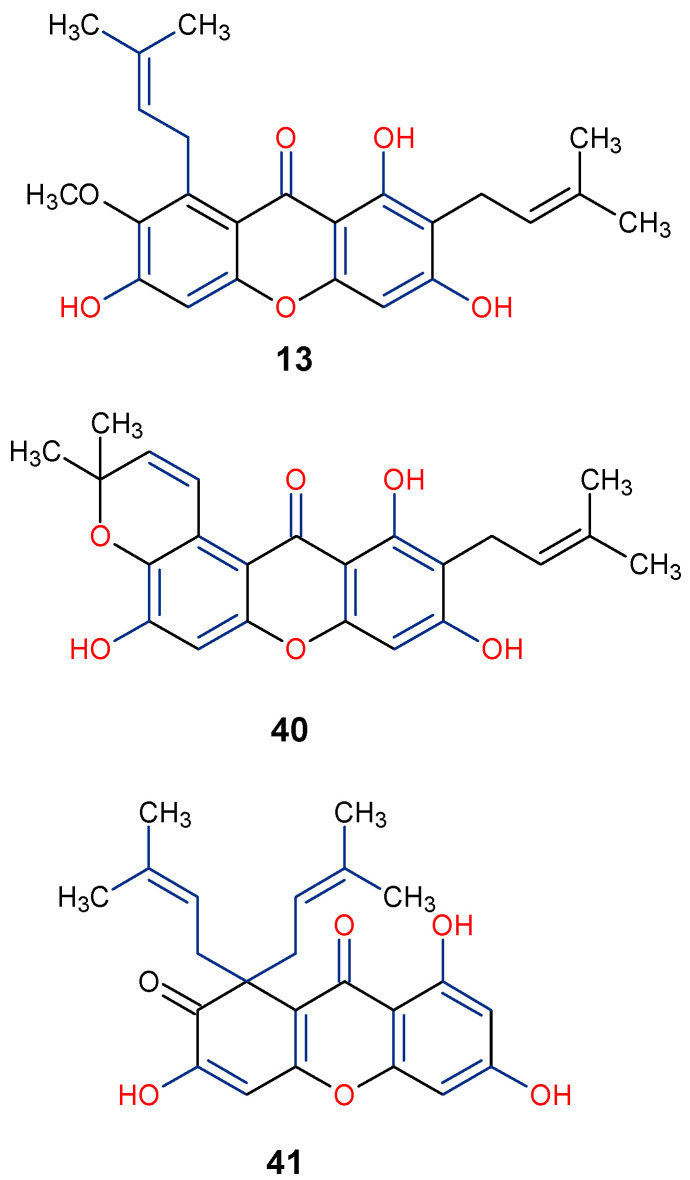
Notable anti-inflammatory xanthones showing their active functional groups in blue and red [80,82].

In the search for natural anti-inflammatory agents, it is critical to look for lead compounds that have one or more of the following pharmacological actions: (1) suppress transcription control of genes encoding enzymes responsible for prostaglandin (PG) biosynthesis or inflammatory cytokines; and (2) prevent the release of PGs, the major chemical mediators in the regulation of inflammation, by directly inhibiting the enzymes responsible for arachidonic acid (AA) and PG biosynthesis, including phospholipase A2 and cyclooxygenase-2 (COX-2) [79]. The anti-inflammatory properties of five xanthone compounds—demethylpaxantonin, patulone, garcinone B, tripteroside, and 1,3,5,6-tetrahydroxyxanthone—purified from an *H. patulum* callus tissue culture were evaluated according to Yamakuni et al. [80]. Two of the xanthones demonstrated considerable activity: patulous (**41**), which inhibits COX-1 activity and A23187-induced PGE2 release, possibly having an anti-inflammatory effect, and garcinone B (**42**), which inhibits both A23187-induced prostaglandin E2 (PGE2) release and lipopolysaccharide (LPS)-induced necrosis factor kappa β (NF-κβ)-dependent transcription. According to these findings, **42** may find use as a neuropharmacological instrument in the investigation of intracellular signaling networks related to inflammation [80]. A great cellular model for researching synoviocyte physiology in connection to the onset and management of rheumatoid arthritis (RA) is the human fibroblast-like synoviocyte rheumatoid arthritis (HFLS-RA) [81]. In an HFLS-RA cell-based assay involving α-Mangostin (**13**), it was discovered that 10 μg/mL of the compound inhibited the nuclear translocation of the transcriptional inflammatory protein, p65, and suppressed the production and activation of important proteins in the NF-κβ pathway to inhibit the inflammatory process [82]. It is worth mentioning that the anti-inflammatory α-Mangostin (**13**), patulone (**40**), and garcinone B (**41**) have their position C-8 substituted with prenyl and pyrano moieties with hydroxyl group at positions C-1, C-3, and C-8 (Figure 9). This underscores the importance of these functional groups on the overall anti-inflammatory activity of xanthones. Further in vivo studies, including mechanistic analysis, would help to establish the anti-inflammatory potency of xanthones and their definitive impact on their structural moieties in combating inflammatory conditions.

### 5.6. Anti-HIV/AIDS Activity

**Figure 10:**
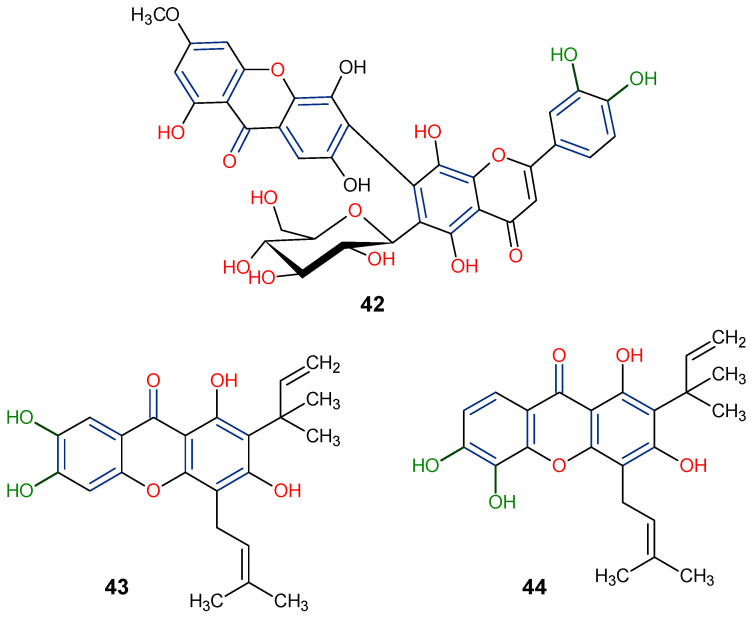
Anti-HIV xanthones from *Swertia franchetiana* [84] and *Maclura tinctoria* [85]. [The functional groups that may be involved in the anti-HIV activity are in multicolor, including the green notation to highlight the catechol group].

Significant advancements have been made over the last three decades in identifying therapeutic approaches for the human immunodeficiency virus (HIV), which causes the acquired immune deficiency syndrome (AIDS). Replication of the virus is inhibited by blocking reverse transcriptase-catalyzed deoxyribonucleic acid (DNA) polymerization from viral ribonucleic acid (RNA), as reverse transcriptase is necessary early in proviral synthesis [83]. Reverse transcriptases are thought to be promising targets for chemotherapy because they may be unique to certain viruses [83]. Natural xanthones have demonstrated action against the human immunodeficiency virus (HIV) in recent times. Many xanthones have secondary therapeutic activities against fungal infections in immune-compromised HIV/AIDS patients in addition to their direct antiviral activity [83]. Swertifrancheside (**42**), a flavone xanthone glycoside (xanthone dimer) from *Swertia franchetiana* has been reported to show a considerable level of activity in inhibiting HIV reverse transcriptase [84]. Two prenylated xanthones, macluraxanthone B (**43**) and macluraxanthone C (**44**), isolated from *Maclura tinctoria* bark have been reported to show considerable anti-HIV activity [85]. After passing an initial anti-HIV screening stage, **43** and **44** demonstrated good potential with EC_50_ values of 1.1–2.0 μg/mL. With IC_50_ levels ranging from 2.2 to 3.7 μg/mL, the catechol functionality (6,7-dihydroxylbenzoyl and 5,6-dihydroxylbenzoyl moieties) of the xanthones appears to give increased HIV inhibitory activity, but they also demonstrate substantial toxicity against CEM-SS host cells [85]. Thus, it may not be far-fetched to attribute the anti-HIV activity of **42**–**44** to the xanthone’s dibenzo-γ-pyrone nucleus, the C-ring (C-1 and C-3) substitution with a hydroxyl group, and the presence of flavone glycoside at position C-6 (Figure 10).

### 5.7. Antidiabetic Activity

The antidiabetic properties of some natural xanthones, such as γ-mangostin (**25**) and mangiferin (**39**), have been potentiated [86]. Mangiferin is a xanthoneglycoside primarily found in the fruits, peels, stembarks, and leaves of *Mangifera indica*, while γ-Mangostin has been reported in the fruits of *Garcinia mangostana* [87,88]. The study showed that xanthones could enhance insulin sensitivity, regulate glucose metabolism, and inhibit oxidative stress and inflammation through the AMP-activated protein kinase (AMPK) and peroxisome proliferator-activated receptors (PPARs) signaling pathways. Based on in vitro studies, it was demonstrated that natural xanthones such as γ-mangostin (**25**) from *Garcinia mangostana* and mangiferin (**39**) from *Mangifera indica* inhibit α-amylase and α-glucosidase enzymes, with IC_50_ values of 3.2 μM and 5.6 μM, respectively, showcasing their potential to improve glucose metabolism [86,89]. Further in vivo studies on **39** in streptozotocin-induced diabetic rats have shown its ability to lower fasting blood glucose levels and improve HDL levels, which may help in the overall glycemic control and lipid profiles [90].

**Figure 11:**
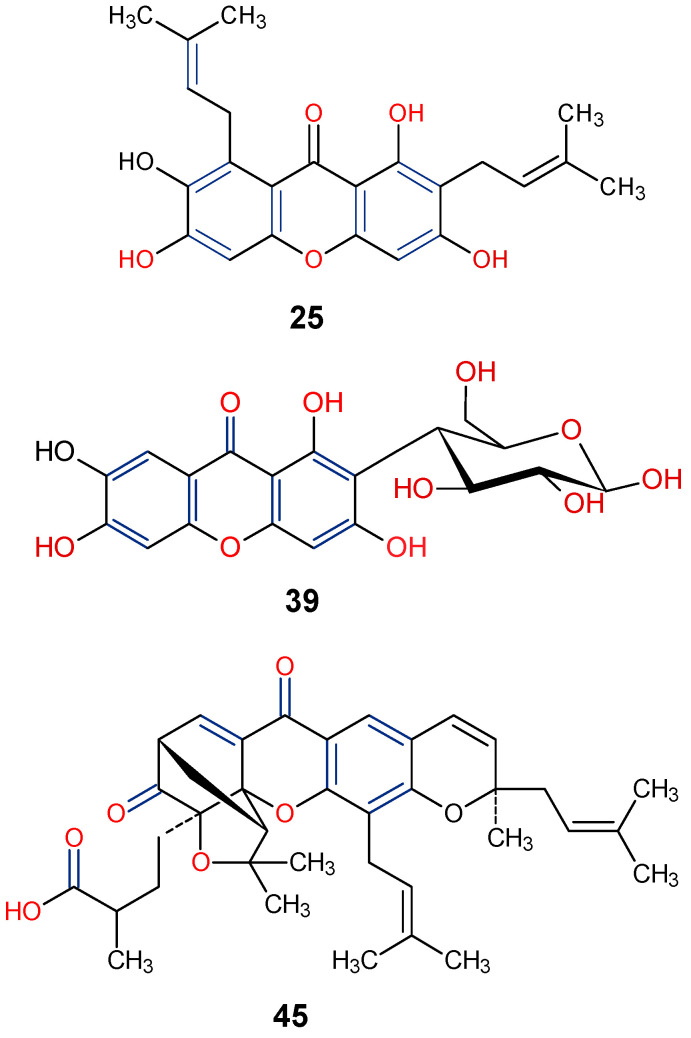
Notable antidiabetic xanthones from *Garcinia mangostana*, *G. hanburyi* and *Mangifera indica* [87,88,91]. [Functional groups in blue and red may be involved in the bioactivity].

Natural xanthones have also been reported to show promising efficacy in diabetes-associated comorbidities such as diabetic retinopathy [91]. For instance, gambogic acid (**45**) isolated from *Garcinia hanburyi*, has been found to reduce high glucose-induced proliferation, migration, and tube formation in choroid-retinal endothelial RF/6A cells [91]. In another related study, gambogic acid (**45**) was found to have a strong antidiabetic retinopathy protective effect by reducing apoptosis and inflammation in human retinal endothelial cells (HRECs) under high glucose environments [92]. From the SAR viewpoint, the key positions C-1, C-3, C-6, C-7, and C-8, are functionalized for enhanced antidiabetic activity of γ-mangostin (**25**), mangiferin (**39**), and gambogic acid (**45**) (Figure 11).

### 5.8. Insecticidal Activity

The fermentation broth of a nonsporulating fungal species, MF6460, isolated from a leaf litter of *Manilkara bidentata* afforded xanthonol (**46**), a dimeric bis-xanthone. The compound exhibited insecticidal and anthelmintic activities against larvae of *Lucilia sericata*, *Aedes aegypti*, and *Haemonchus contortus* with lethal doses (LD_90_) of 33, 8, and 50 µg/mL, respectively [93]. Simaomicin α (LL-D42067 α) is a polycyclic xanthone isolated from the fermentation broth of an actinomycete strain of *Actinomadura madurae* [94]. Simaomicin α (**47**) demonstrated remarkably higher antimalarial activities compared to chloroquine, artemisinin, and artemether when evaluated against drug-resistant (K1) and drug-sensitive (FCR3) *Plasmodium falciparum* strains with IC_50_ values of 0.045 and 0.0097 ng/mL, respectively. It also inhibited the growth of *P. falciparum* in a time and concentration-dependent manner [95]. In another study, a polycyclic xanthone, MDN-0185 (**48**), isolated from *Micromonospora* sp. CA-256353, exhibited in vitro antiplasmodial activity with an IC_50_ of 9 nM against *Plasmodium falciparum* 3D7 parasites [96]. Lastly, plants in the genus *Garcinia* produce some caged-xanthone derivatives, notably Gambogic acid (**45**) and Cluvenone (**49**), with both exhibiting notable antimalarial activities with EC_50_ values of 0.28 ± 0.03 and 0.75 ± 0.03 μM, respectively [97].

Based on the discussed biological activities of natural xanthones with their structural features, it can be summarized that xanthones are a unique class of compounds with a wide range of biological applications owing to their tricyclic dibenzo-γ-pyrone nucleus. It is worthy of mention that some xanthones from the mangosteen plant (*G. mangostana*), are known to display dual bioactivity. For instance, garcinone E (50) is a potent dual inhibitor of epidermal growth factor receptor (EGFR) and vascular endothelial growth factor receptor 2 (VEGFR2) [98]. Also, α-mangostin (13), γ-mangostin (25), and 8-deoxygartanin (31) showed considerable multitarget actions against the digestive enzymes, α-amylase (IC_50_ = 33.3 µM), α-glucosidase (IC_50_ = 69.2 µM) and pancreatic lipase (164.4 µM) [99]. These enzymes are known to play an important role in the metabolism of carbohydrates and lipids Thus, the mangosteen xanthones could further be exploited in the search for attractive therapeutic targets for the treatment of type 2 diabetes and obesity Therefore, further pharmacological assessment of these xanthones may be worthwhile.

**Figure 12:**
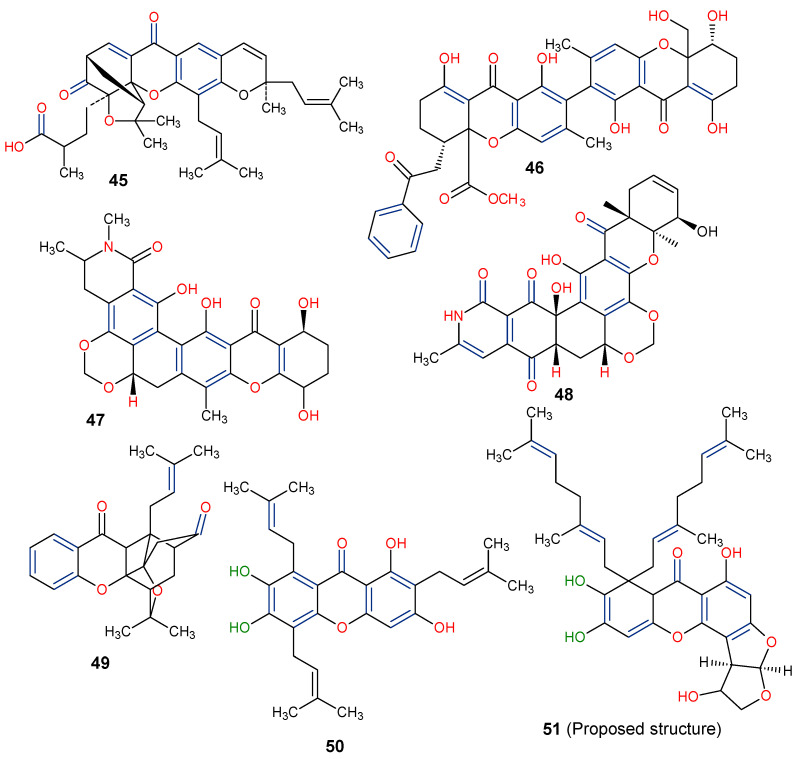
Chemical structures of some xanthones showing those with insecticidal activities (**45**–**49**) [93,94,95,96,97] and a proposed structure for possible biological evaluation (**50**). [The functional groups that may be involved in the bioactivity are in multicolor, including the green notation to highlight the catechol group].

Finally, the distinct chemical scaffold of xanthones, such as prenylation at the C-8 position, including the geranyl (C-10) substituent, the presence of hydroxyl group at positions C-1, C-6, and C-7, as well as the attachment of furan ring to the C-ring, may confer interesting biological activities on xanthones. Thus, having compounds with such structural construct, as in the proposed 8,8-Bis-(3,7-dimethyl-octa-2,6-dienyl)-1,6,9,10-tetrahydroxy-1,2,3a,7a,8,12c-hexahydro-furo[3′,2′:4,5]furo[2,3-c]xanthen-7-one (51) (Figure 12), it is expected that xanthones and their derivatives will continue to attract much interest in drug discovery.

## 6. Conclusions and Future Prospects

Naturally occurring xanthones have been discussed through this review to showcase their natural abundance and distinct biosynthetic pathways, giving rise to unique chemical scaffolds with notable biological activities, including antifungal, antibacterial, anticancer, coagulant, antioxidant, anti-inflammatory, anti-HIV/AIDS, antidiabetic, and insecticidal activities. Guttiferae, Gentianaceae, and Polygalaceae are among the higher plant families with abundant xanthone sources, while Aspergillaceae is prominent among the families of lower plants containing this unique chemical class. From the structure–activity relationship viewpoint, the effect of prenylation, oxygenation (hydroxyl group), and glycosylation, as well as furan and pyran substitutions of the dibenzo-γ-pyrone nucleus of xanthones, on the biological activities have been discussed. Therefore, having xanthones characterized by these chemical substituents at positions C-1, C-3, C-6, and C-8 may help to generate more active analogs as possible drug candidates. Among the promising xanthones highlighted in this study are γ- and α-mangostins, norathyriol, mangiferin, and isomangiferin, sterigmatocystin, while the structure, 8,8-Bis-(3,7-dimethyl-octa-2,6-dienyl)-1,6,9,10-tetrahydroxy-1,2,3a,7a,8,12c-hexahydro-furo[3′,2′:4,5]furo[2,3-c]xanthen-7-one, which may offer greater biological effect, has been proposed though this study. However, the available biological data on xanthones is still limited. There is, therefore, a need for further investigation of the promising xanthones through efficacy and safety studies. With this, there would be enough in vivo and clinical data to sufficiently decipher the specific pharmacological actions of the promising compounds and their molecular mechanism of action for lead optimization.

## Figures and Tables

**Table 1:** Classification of naturally occurring xanthones.

Parent Class	Structural Example	Natural Source (Family)	Reference
Based on the level of oxidation of the C-ring			
Monomers 1 (Xanthones)	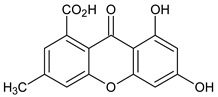 **6,8-Dihydroxy-3-methylxanthone-1-carboxylic acid**	*Penicillium oxalicum* (Trichocomaceae)	[37]
	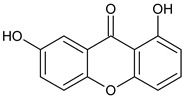 **1,7-Dihydroxyxanthone** 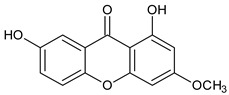 **Gentisin**	*Garcinia lancilimba* (Guttiferae)	[38]
Monomer 2 (Dihydroxanthones)	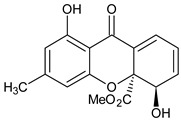 **Nidulalin A**	*Anixiella micropertusa* (Sordariaceae)	[39]
	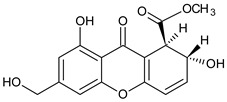 **Dihydroxanthone AGI-B4**	*Diaporthe* species (Diaporthaceae)	[40]
Monomer 3 (Tetrahydroxanthones)	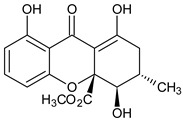 **Blennolide B** **(Hemi-secalonic acid E)**	*Blennoria* species (Coleosporiaceae)	[41]
	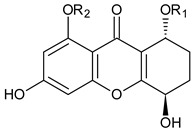 **Amarellin A: R_1_ = Glc, R_2_ = H** **Amarellin B: R_1_ = Xyl, R_2_ = H** **Amarellin C: R_1_ = H, R_2_ = Glc**	*Gentianella amarella* ssp. *Acuta* (Gentianaceae)	[42]
Monomer 4 (Hexahydroxanthones)	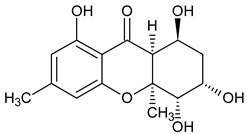 **Monodictysin A**	*Monodictys putredinis* (Thrombiaceae)	[43]
	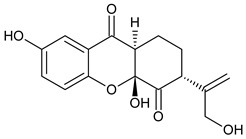 **Applanatin A**	*Ganoderma applanatum* (Ganodermataceae)	[44]
Dimers and Heterodimers 1 (Xanthones)	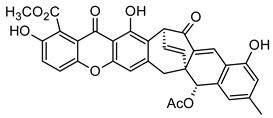 **Acremoxanthone A**	*Acremonium* sp. (Hypocreaceae)	[45]
	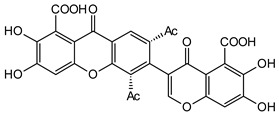 **Vinaxanthone**	*Penicillium glabrum* (Trichocomaceae)	[35]
Dimers and Heterodimers 1 (Dihydroxanthones)	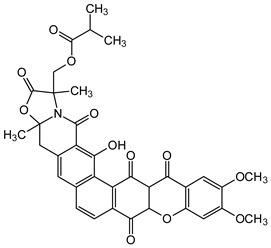 **Citreamicin β**	*Micromonospora citrea* (Micromonosporaceae)	[46]
Dimers and Heterodimers 3 (Tetrahydroxanthone)	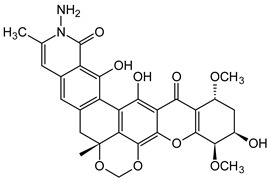 **Actinoplanone C**	*Actinoplanes* species (Micromonosporaceae)	[47]
Based on the level of oxygenation/type of ring residue			
Simple oxygenated xanthones (mono-oxygenated)	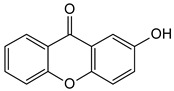 **2-Hydroxyxanthone**	*Mammea americana* (Calophyllaceae)	[48,49]
	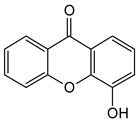 **4-Hydroxyxanthone**	*Calophyllum inophyllum* (Calophyllaceae)	
Di-oxygenated	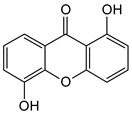 **1,5-Dihydroxyxanthone**	*Mesua ferrea* (Calophyllaceae)	[50]
Tri-oxygenated	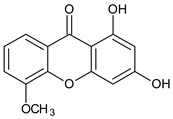 **1,3-Dihydroxy-5-methoxyxanthone**	*Cudrania fruticose* (Moraceae)	[51]
Tetra-oxygenated	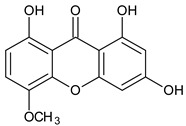 **1,3-Dihydroxy-5methoxyxanthone**	*Swertia nervosa* (Gentianaceae)	[52]
Penta-oxygenated	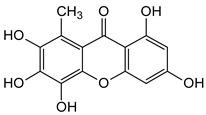 **Anomalin B**	*Swertia purpurascens* (Gentianaceae)	[53]
Hexa-oxygenated	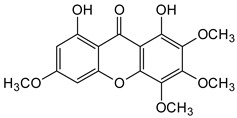 **1,8-Dihydroxy-2,3,4,6-tetramethoxyxanthone**	*Centaurium erythraea* (Gentianaceae)	[54]
Prenylated and related xanthones	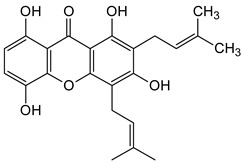 **Gartanin** 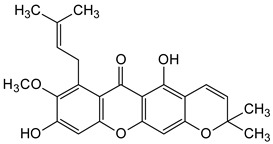 **9-Hydroxycalabaxanthone**	*Garcinia mangostana* (Guttiferae)	[3]
Bisxanthones	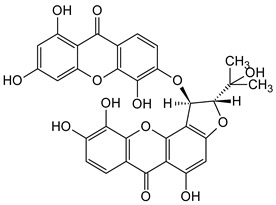 **Jacarelhyperols D**	*Hypericum japonicum* (Guttiferae)	[55]
Xanthonolignoids	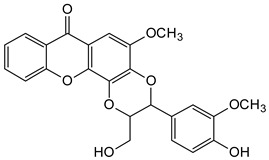 **Kielcorin**	*Kielmeyera coriacea* (Guttiferae)	[56]
Other miscellaneous xanthones	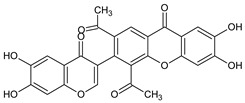 **Xanthonopyrone, SPF-3059-26**	*Penicillium erubescens* KUFA 0220 (Aspergillaceae) isolated from the marine sponge *Neopetrosia* sp.	[57]
	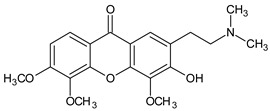 **2-(2-(Dimethylamino)ethyl)-3-hydroxy-4,5,6-trimethoxy-9H-xanthen-9-one**	*Caulophyllum robustum* (Berberidaceae)	[58]
	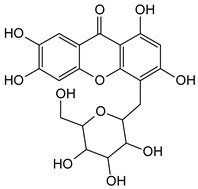 **Isomangiferin**	*Mangifera indica* (Anacardiaceae)	[59]

## Data Availability

Not applicable.
